#  A Multi Residue GC-MS Method for Determination of 12 Pesticides in Cucumber

**Published:** 2016

**Authors:** Azadeh Nasiri, Maryam Amirahmadi, Zahra Mousavi, Shahram Shoeibi, Alireza Khajeamiri, Farzad Kobarfard

**Affiliations:** a *Department of Pharmacology and Toxicology, Faculty of Pharmacy, Pharmaceutical Sciences Branch, Islamic Azad University,Tehran-Iran (IAUPS).*; b *Food and Drug Laboratories Research Center, Ministry of Health and Medical Education, Tehran, Iran.*; c *Food and Drug Control Laboratories, Food and Drug Deputy, Ministry of Health and Medical Education, Tehran, Iran. *; d *Policy University, Tehran, Iran. *; e *Department of Medicinal Chemistry, School of Pharmacy, Shahid Beheshti Medical University, Tehran, Iran.*

**Keywords:** Pesticide, Spiked calibration curve, GC/MS, Cucumber, Multi residue, Iran

## Abstract

Cucumber is one of the main vegetables in Iranian food basket. A wide range of pesticides are used for crops protection during the cultivation of vegetables such as cucumber due to heavy pest infestation. Analysis of pesticide residues in food and other environmental commodities have become essential requirement for consumers, producers, and food quality control authorities. This study was aimed at determination of pesticides residues in cucumber as a main vegetable in Iranian food basket.

A reliable, rapid and accurate method based on spiked calibration curves and modified QuEChERS sample preparation was developed for determination of 12 pesticide residues in cucumber by gas chromatography-mass spectrometry (GC/MS). The use of spiked calibration standards for constructing the calibration curve substantially reduced adverse matrix-related effects.

The recovery of pesticides at 5 concentration levels (n = 3) was in the range of 80.6-112.3. The method was proved to be repeatable with RSD lower than 20%. The limits of detection and quantification for all pesticides were <10 ng/g and <25 ng/g, respectively. The developed method was used for simultaneous determination of the selected pesticides in 60 greenhouse and garden cucumber samples. Among the 60 analyzed samples, 41.7% of them were contaminated with pesticide residues which 31.7% of samples had pesticide residues lower than maximum residue limit and 10% of samples had residue higher than maximum residue limit.

## Introduction

Cucumber «*cucumas sativus*» is a frequently consumed vegetable in Iranian daily food basket (1). The increasing public concern about pesticide contamination of food and the environment in recent years has increased the demand for broader and stricter pesticide monitoring. Therefore, it is necessary to develop rapid, reliable and effective analytical methods for the simultaneous determination of the residues of pesticides in order to obtain accurate information about the types and quantity of the pesticides used, to protect the consumers and guarantee the safety of agricultural products (2). The authorities are criticized for monitoring too few samples, which stems from the high cost, complexity, and time-consuming sample preparation procedures involved in current chromatographic methods for pesticides analysis (3). 

Over the past decades, approaches to trace level determination of pesticides have moved away from the use of GC with selective detectors including electron capture detection (ECD) (4-6) and nitrogen phosphorus detection (NPD) (7), to mass spectrometer (MS) detectors which are more sensitive and selective (8). The use of mass spectrometry, with its information-rich content and explicit confirmation, is recommended for monitoring pesticide residues in the entire world (9-13). Generally, the complex matrix of agricultural products adversely affects analysis precision, and it is necessary to remove the matrix interference by sample pre-treatment, such as extraction and clean-up steps (14-16). 

Conventionally, simultaneous analysis of pesticide residues from different classes in foods is a time-consuming process that often entails several post-extraction clean up steps prior to the analysis. A simple, safe, cheap and high sample throughput method named QuEChERS has been recently proposed for the analysis of pesticide residues (17-21).

This paper presents a rapid multi-residue method of analysis based on QuEChERS extraction procedure using spiked calibration curve to simultaneously determine and confirm 14 pesticide residues in cucumber. The selected pesticides included GC-amenable pesticides, those for which MRL is issued by Institute of Standards of Iran (22). 

In order to overcome the adverse matrix-related effects, it was decided to prepare the calibration curves by spiking blank cucumber samples with certain amounts of pesticides and constructing the calibration curve using these spiked samples. The list of selected pesticides along with some of their physico-chemical properties are presented in Table 1.

## Materials and Methods

*Reagent*

All pesticides standards were purchased from Dr. Ehrenstorfer Co. (Augsburg, Germany). All organic solvents, intended for extraction, were at least LC grade and purchased from Merck (Darmstadt, Germany). Primary secondary amine (PSA) and graphite carbon black (GCB) were purchased from Supelco (Bellefonte, USA). Bulk quantities of NaCl were obtained from Merck (Darmstadt, Germany). Anhydrous MgSO_4_ was obtained from SIGMA-Aldrich CO. (Japan). The MgSO_4_ was baked for 5 h at 500 ºC in a furnace to remove phthalates and residual water. 

*Apparatus*

An Agilent Technologies 6890 Network GC System chromatograph (Wilmington, USA) with a (single quadrupole) SQ detector and equipped with an Agilent 7683B autosampler (Agilent technologies, USA) was used. A HP-5 capillary column (30 m×0.25 mm I.D., 1 μm film thickness) 

Real Samples**:** sixty cucumber samples were collected from Tehran Central Market fruits and vegetables in 2011. Cucumber samples from 4 different cities were prepared that 43 of them were greenhouse products and the others were garden cucumber. In this study, in addition to monitoring 12 pesticides residues in cucumber, the pesticides residues in greenhouse cucumbers were compared with those of garden cucumbers.

*Calibration standards*

Individual stock standard solutions (1 mg/mL) were prepared in ethyl acetate and stored in the dark at −20 °C. Prior to their use, they were kept for 1 h at ambient temperature. A mixed stock standard solution of pesticides was prepared in ethyl acetate at 10 μg /mL with respect to each pesticide. Spiked calibration curves at 7 levels of 10, 25, 50, 100, 200, 300 and 500 ng/g in triplicates were prepared by addition of 10 μL, 25 μL, 50 μL, 100 μL, 200 μL, 300 μL and 500μL of mixed standard stock solution, respectively, to 10 g portions of blank cucumber samples in each case. 

A stock solution of triphenylmethane (TPM) in ethyl acetate at concentration of 1 mg/mL was used as internal standard and an aliquot of 10 μL of TPM solution in ethyl acetate was added to the spiked cucumber sample. The samples so obtained were treated as described in section 2.4. 

*Sample preparation*

An aliquot of 10 μL of internal standard solution (1000 mg/L) was added to 10 g of blended blank cucumber sample in a 50 mL falcon tube and after being left for 1h at ambient temperature in dark, 20 mL (ethanol, toluene, 50%:50%) was added. The mixture was mixed at high speed with vortex mixer for 1min. One gram of NaCl and 2 grams of activated anhydrous MgSO_4 _was added to the mixture, and mixing was continued for an additional 60 s. The mixture was centrifuged for 5 min at 5000 rpm at -5 ^◦^C. The supernatant was transferred to a 15 mL falcon tube containing 2 g MgSO_4_ and 300 mg PSA and 200 mg GCB. After shaking for 1 min and centrifugation for 5 min at 5000 rpm at -5 ^◦^C, 4 mL of supernatant was transferred to a 5 mL vial and evaporated to dryness under a gentle stream of nitrogen gas. The residue was reconstituted by toluene to obtain 1 mL solution, and after shaking for 3 min, 2 μL of the solution was injected into gas chromatograph. 

*Recovery studies*

For recovery determination, spiked blank cucumber samples at concentration levels of 30, 60, 150, 250, 350 ng/ g were prepared in triplicates and they were kept for 1 h at ambient temperature prior to their use and then treated according to the procedure described in section 2.4. The recoveries were calculated using the calibration curves constructed using spiked samples. 

*GC/MS analysis*

The GC/MS was employed with helium as the carrier gas at a constant flow of 1 mL/min. The oven temperature started at 75 °C and remained at this temperature for 3 min increasing to 120 °C at 25 °C/min ramp rate and then increased to 300 °C at 5 °C/min ramp, holding at 300 °C for 11 min. Injection port was adjusted at 250 °C and splitless injection mode was used.

After acquisition of the total ion chromatogram for the mixed stock standard solutions in scan mode, peaks were identified by their retention time and mass spectra. The most abundant ion that showed no evidence of chromatographic interference and had the highest signal-to-noise ratio was selected for quantification purposes. 

*Quantitation*

The concentrations of pesticides were determined by intrapolation of the relative peak areas for each pesticide to internal standard peak area in the sample on the spiked calibration curve. In order to compensate for losses during sample processing and instrumental analysis, internal standard (TPM) was used. 

## Results

*Gas chromatographic determination*

Analysis was performed in the SIM mode based on the use of one target and two or three qualifier ions. Pesticides were identified according to their retention times and target and qualifier ions. The quantitation was based on the peak area ratio of the targets to that of internal standard. Table 2. summarizes pesticides studied with their diagnostic and quantification ions used in SIM mode in this study.

*Method validation*

*Linearity of the calibration curves*

The twelve pesticides showed linearity in SIM mode. Linear spiked calibration curves for all the interest pesticides were obtained with correlation factors >0.996. The Calibration data (equation and regression coefficient) of 12 pesticides in spiked cucumber calibration curves is showed in Table 3. 

*LOD and LOQ*

Limits of detection (LOD) and limits of quantification (LOQ) of the proposed method were measured in spiked samples and calculated by considering a value 3 and 10 times that of background noise, respectively. The LODs and LOQ for all the pesticides were ≤10 ng/g and ≤25 ng/g respectively. 

*Accuracy and Precision*

Table 4. presents the recovery and repeatability for five concentration levels of pesticides. The recovery of pesticides at 5 concentration levels triplicates was in the range of 80.6-112.3%. In terms of repeatability, the majority of the pesticides gave RSD<20%. The recoveries and repeatabilities are in accordance with the criteria set by SANCO Guideline (23).

## Discussion

The major source of inaccuracy in pesticide residue analysis by GC-MS, especially in food, is related to the presence of interfering components in the sample, the so-called ‘‘matrix effect’’. In other words, in conventional gas chromatographic analysis, such as the analysis of pesticide residues in foods, co-extracted matrix components may be problematic in obtaining the true data. (13).

Theoretically, elimination of matrix components or active sites in the injection port would surmount the matrix-induced enhancement effect; but, complete and permanent GC system deactivation or comprehensive sample clean up is practically impossible (24).

**Figure 1:**
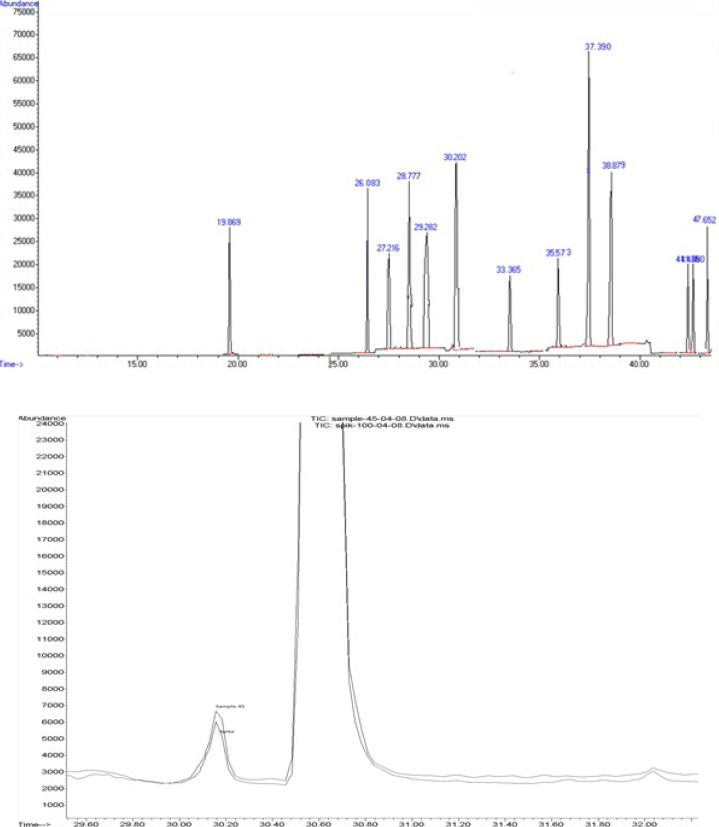
An overlaid GC-MS-SIM chromatogram of (a) cucumber sample spiked at 100 ng/g of diazinone (rt=26 min), primicarb (rt=27.2 min) and chlorpyrifos (rt=30.2 min) and (b) contaminated cucumber sample with chlorpyrifos

**Table 1.** Physicochemical properties of the selected pesticides

**Compound**	**Structural group**	**M.F.**	**M.W.**	**M.P. (ºC )**
Diazinon	Organophosphorus	C12H21N2O3PS	304.35	<120 °C
Chlorpyrifos	Organophosphorus	C9H11CL3NO3PS	350.59	41.5-43.5 °C
Pirimiphos-methyl	Organophosphorus	C11H20N3O3PS	305	15 °C
Beta-Endosufan	Organochlorous	C9H6CL6O3S	406.93	207-209 °C
Alpha-Endosulfan	Organochlorous	C9H6CL6O3S	406.92	106 °C
Fenvalerate1,2	Pyrethroid	C25H22CLNO3	419.91	<25 °C
Primicarb	Carbamat	C11H18N4O2	238.29	90.5 °C
Carbaryl	Carbamat	C12H11NO2	201.22	142.2 °C
Propargite	Sulfite ester	C19H26O4S	350.5	47 °C
Fenpropathrin	Pyrethroid	C22H23NO3	349.4	47 °C
Permethrin1,2	Pyrethroid	C21H20Cl2O3	391.29	34 °C
Metalaxyl	Acylalanin	C15H21NO4	279.34	72 °C

**Table 2:** The retention time, diagnostic ions and selected quantification ion for the target pesticides and internal standard

**No.**	**Compound**	**Diagnostic ions (m/z)**	**Quantification ions (m/z)**	**Retention time (min)**
**1**	Carbaryl	144, 115.1, 145.1, 116.1	144.1	19.869
**2**	Diazinon	304, 276.1, 179,1	304.1	26.083
**3**	Pirimicarb	238.2, 166.1, 138	166.1	27.216
**4**	Metalaxyl	206.1, 249,234	206.1	28.777
**5**	Pirimiphos methyl	305, 290.1, 276	290.1	29.282
**6**	Chlorpyrifos	314, 257.8, 316	314.0	30.202
**7**	Triphenyl methan	236.9, 264.9, 338.9	244.1	30.648
**8**	Alpha-Endosulfan	236.9, 264.9, 338.9	236.9	33.365
**9**	Beta-Endosulfan	339.1, 264.9, 236.9	236.9	35.573
**10**	Propargite	350.2, 173.1, 201.1	350.2	37.390
**11**	Fenpropathrin	265.1, 208, 349.2	265.1	38.879
**12**	Permethrin 1	183.1,163.1,184.1	183.1	42.233
**13**	Permethrin 2	183.1,163.1,184.1	183.1	42.500
**14**	Fenvalerate 1	419.2, 225.1, 167.1	167.1	47.256
**15**	Fenvalerate 2	419.2, 225.1, 167.1	167.1	47.911

**Table 3.** Calibration data (equation and regression coefficient) of 12 pesticides in spiked cucumber calibration curves.

**Compound**	**Equation**	**Regression Coefficient**
Carbaryl	y = 0.1899x – 0.016	0.999
Diazinon	y = 0.1482x + 0.0003	0.999
Pirimicarb	y = 0.6757x - 0.0012	0.999
Metalaxyl	y = 0.2803x + 0.0056	0.998
Pirimiphos-methyl	y = 0.3587x + 0002	0.998
Chlorpyrifos	y = 0.1978x - 0.0001	0.999
Alpha-Endosulfan	y = 0.11797x - 0.0023	0.999
Beta-Endosulfan	y = 0.0568x + 0.0003	0.996
Propargite	y = 0.0965x - 0.0003	0.999
Fenpropathrin	y = 0.1351x+ 0.0006	0.999
Permethrin 1,2	y = 0.7747x + 0.0029	0.999
Fenvalerate 1,2	y = 0.1819x + 0.0017	0.997

**Table 4:** Average recoveries (%) and relative standard deviations (%) of pesticides obtained by GC-MS analysis of cucumber samples at 5 spiking levels (n=3).

Compound	Average recovery (%) (n=3)					Total Average recovery (%) (n=3)	Range of RDS (%)
	30ng/g	60ng/g	150ng/g	250ng/g	350ng/g		
Carbaryl	101.3	103.2	112.4	78.1	87.3	96.5	5.4-16.8
Diazinon	90.7	108.5	112.6	98.1	102.4	102.5	1.8-11.2
Pirimicarb	112.4	117.8	114.3	102.7	108.7	111.2	10.1-19.9
Metalaxyl	75.8	80.2	90.5	82.5	84.1	82.6	0.6-24.1
Pirimiphos methyl	88.4	108.2	118.2	92.2	97.6	100.9	7.1-23.3
Chlorpyrifos	97.2	102.8	104.1	96.2	96.6	96.6	2.9-5.6
Alpha-Endosulfan	116.5	88.4	68.1	77.5	80.7	85.7	0.9-9.9
Beta-Endosulfan	112.4	117.8	114.3	102.7	108.7	80.6	3.3-9.3
Propargite	75.8	80.2	90.5	82.5	84.1	112.1	1.7-22.8
Fenpropathrin	88.4	108.2	118.2	92.2	97.6	91.1	2.7-19.1
Permethrin 1, 2	80.1	95.6	101.3	92.5	96.7	96.8	3.3-11.0
Fenvalerate1, 2	120.6	117.8	97.7	112.5	121.3	112.3	3.7-11.8

**Table 5:** Pesticide residues detected on 60 cucumber samples, Iran, 2011.

**MRL** **µg/g**	**Range** **ng/g **	**Mean of Pesticide residue ng/g**	**Pesticide type (number)**	** Samples condition (detected vs not-detected)**	**Samples (number)**
		< LOD	12 pesticide understudy	Pesticide not-detected (26)	Green House (43)
	40.3	40.3	Fenpropathrin (1)	Pesticide detected (17)	
1	27.7-34.8	31.25	Alpha-Endosulfan(2)		
1	7.6-17.8	12.7	Beta-Endosulfan (2)		
0.5	7.3-194.1	57.58	Metalaxyl (6)		
0.05	66.4-148.2	97.13	Chlorpryfos (6)		
		< LOD	12 pesticide understudy	Pesticide not-detected (8)	Garden (17)
0.5	36	36	Propargite (1)	Pesticide detected (9)	
0.5	40-87.6	65.87	Metalaxyl (8)		

There are a number of approaches for preventing, reducing, or compensating for the occurrence of matrix effects (13, 21) including the application of alternative calibration methods including the use of (A) matrix-matched calibration method, (25-27), (B) standard addition method, (C) isotopically labeled internal standards (not feasible in multiresidue pesticide analysis due to their unavailability or high price) and (D) usage of analyte protectants (21).

In the present study, we used spiked calibration curves approach to overcome the problems caused by the matrix. In this approach, calibration curves are constructed by the addition of standard solution to blank cucumber samples and these samples are subjected to the same sample preparation procedure which is intended to be used for unknown samples. This way, the standard sample matrices will have the same composition as the unknown samples and therefore the effect of matrix is reflected in both standards and unknown samples. The calibration curve is constructed using these spiked calibration standards and it is easily used to calculate the concentration of analyte(s) in unknown sample without being concerned about the matrix effects. Figure 1. represents an overlaid GC-MS-SIM chromatogram of a cucumber sample spiked at 100 ng/g of diazinone (rt = 26 min), primicarb (rt = 27.2 min) and chlorpyrifos (rt = 30.2 min) and a contaminated cucumber sample with chlorpyrifos.

The recoveries and repeatabilities were in accordance with the criteria set by SANCO Guideline (23). 

It appears that the proposed spiked calibration curve method is a proper approach for elimination of matrix effects in pesticide residues analysis. The method could be considered as an alternative method along with matrix-matched standards, standard addition method, isotopically labeled internal standards and usage of analyte protectants, for determination of various classes of pesticide residues. It is rapid, simple, sensitive, selective and rugged.

A comparison of the results for greenhouse cucumbers with the results obtained for garden cucumbers showed that the residues were different in both identity and quantity (Table 5).

In greenhouse cucumber samples Fenpropathrin, Alpha-Endosulfan, Beta-Endosulfan, Metalaxyl and Chlorpryfos were detected while in garden cucumbers, Propargite and Metalaxyl were detected. The diversity and quantity of pesticides in greenhouse cucumber were higher than garden cucumbers. It seems that the high levels of pesticides residues in greenhouse cucumbers is due to their particular product conditions and higher utilization of pesticides to control pathogens in cultivation of greenhouse cucumbers. 

## Conclusion

A simple and rapid method was developed to determine 14 pesticide residues in cucumber, a frequently used vegetable in Iranian food basket. The method which consists of a QuEChERS simple sample preparation procedure and GC-SQ-MS-SIM analysis showed a high sensitivity and confirmatory power necessary for the determination of pesticide residues at the levels of maximum residue limits (MRLs) issued in Iran for cucumber. The developed method has the advantage of using spiked calibration curves that minimizes the matrix interferences leading to higher accuracy for pesticides analyses. Among the 60 analyzed cucumber samples, 41.7% of them were contaminated with pesticide residues; 31.7% of the samples had pesticide residues lower than maximum residue limit and 10% of samples had residue higher than maximum residue limit indicating the need for awareness about the greenhouse cucumber production.
